# Baseline monocyte SGLT2 mRNA expression and changes in the fibrosis-4 index after luseogliflozin treatment in participants with type 2 diabetes and MASLD

**DOI:** 10.1007/s13340-026-00941-2

**Published:** 2026-10-31

**Authors:** Hideyuki Okuma

**Affiliations:** Department of Diabetes and Endocrinology, Nerima Hikarigaoka Hospital, Nerima-Ku, 179-0072 Japan

**Keywords:** Type 2 diabetes, Metabolic dysfunction-associated steatotic liver disease, Sodium-glucose cotransporter 2, Monocytes, Fibrosis-4 index

## Abstract

**Background:**

Metabolic dysfunction-associated steatotic liver disease (MASLD) is common in type 2 diabetes. We investigated the association between monocyte mRNA expression of sodium-glucose cotransporter 2 (SGLT2), the molecular target of luseogliflozin, and changes in the fibrosis-4 (FIB-4) index during treatment with this agent.

**Methods:**

This single-center retrospective observational study used prospectively collected peripheral blood monocyte samples from 82 participants with type 2 diabetes and MASLD who had received luseogliflozin for at least 6 months. Baseline monocyte SGLT2 mRNA expression was measured using quantitative real-time polymerase chain reaction. Clinical and laboratory parameters, including FIB-4 index, were evaluated at baseline and 6 months after luseogliflozin initiation. Changes were calculated as the 6-month value minus the baseline value; therefore, negative values indicated reductions.

**Results:**

After 6 months, waist circumference, hemoglobin A1c, liver enzyme levels, high-sensitivity C-reactive protein, and the FIB-4 index decreased significantly. Baseline monocyte SGLT2 mRNA expression was negatively correlated with the change in the FIB-4 index, indicating that higher baseline expression was associated with a greater reduction. In multiple regression analyses, natural-log-transformed baseline monocyte SGLT2 mRNA expression remained associated with the change in the FIB-4 index after adjustment for baseline clinical variables.

**Conclusions:**

Higher baseline monocyte SGLT2 mRNA expression was associated with a greater reduction in the FIB-4 index after luseogliflozin treatment. This finding should be interpreted as an exploratory association between a cell-specific transcriptomic feature and changes in a fibrosis-related index, rather than as evidence of functional SGLT2 signaling in monocytes or histological fibrosis regression.

**Graphical abstract:**

**Figure:**
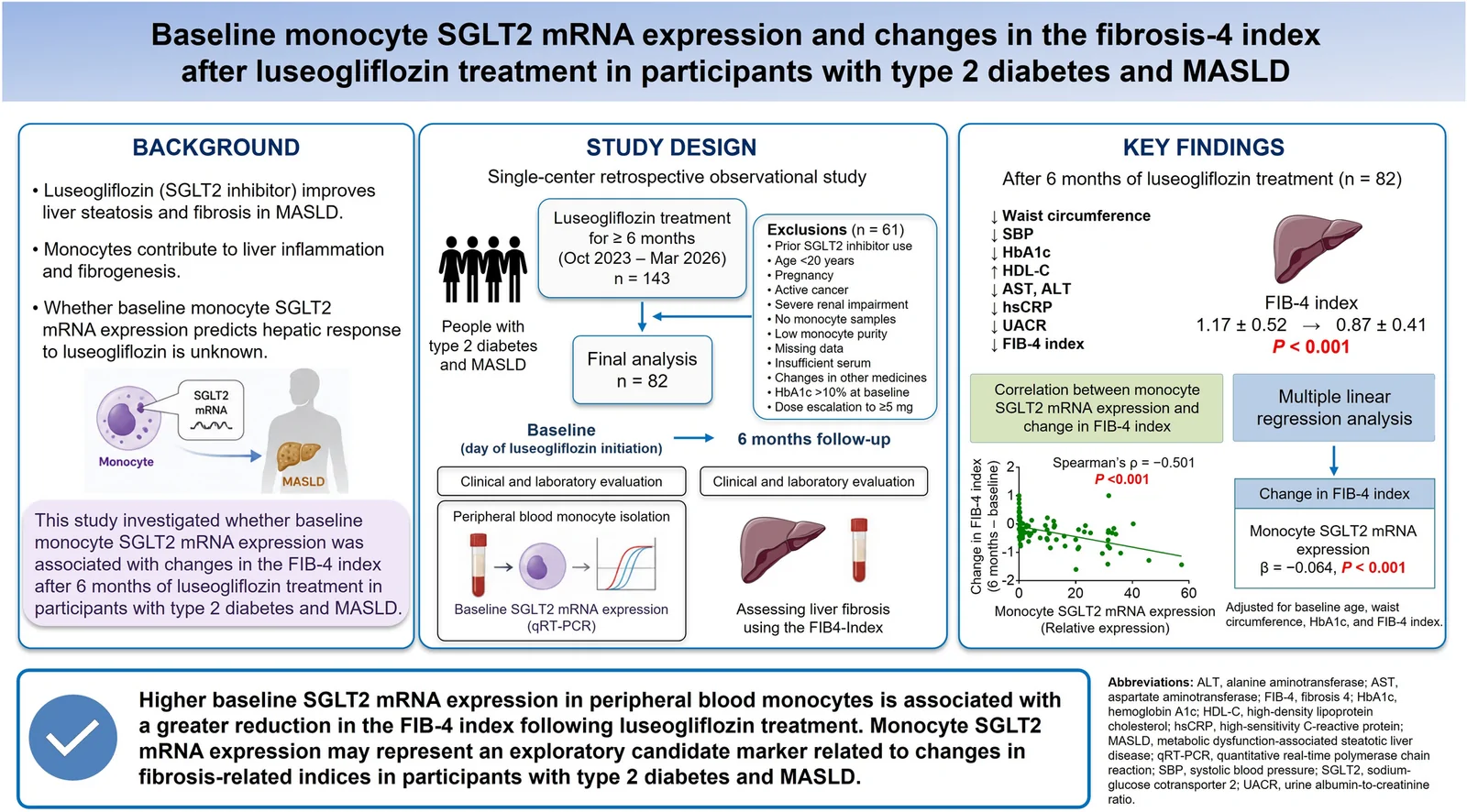

**Supplementary Information:**

The online version contains supplementary material available at https://doi.org/10.1007/s13340-026-00941-2.

## Introduction

Metabolic dysfunction-associated steatotic liver disease (MASLD) is highly prevalent among patients with type 2 diabetes and is associated with obesity, insulin resistance, and cardiometabolic comorbidities [1]. Liver fibrosis is a major determinant of clinical outcomes in MASLD, making non-invasive fibrosis assessment important in clinical practice [2]. Sodium-glucose cotransporter 2 (SGLT2) inhibitors reduce renal glucose reabsorption and provide cardiovascular and renal benefits beyond glycemic control [3]. Evidence suggests that SGLT2 inhibitors improve hepatic steatosis and fibrosis in MASLD [4]. Consistent with these findings, luseogliflozin, a selective SGLT2 inhibitor, has been reported to reduce intrahepatic fat accumulation and improve liver-related biochemical parameters in patients with type 2 diabetes and MASLD [5, 6]. However, markers associated with changes in liver-related parameters after luseogliflozin treatment remain unclear.

Monocytes and macrophages contribute to MASLD progression from steatosis to steatohepatitis and fibrosis. Circulating monocytes are recruited to the liver and differentiate into macrophages, promoting hepatic inflammation and fibrogenesis [7]. C–C chemokine receptor type 2 (CCR2)-dependent monocyte recruitment promotes steatohepatitis, and therapeutic inhibition of this pathway attenuates steatohepatitis and liver fibrosis [7, 8]. Consistently, we recently reported that cholesterol accumulation in peripheral blood monocytes was associated with the fibrosis-4 (FIB-4) index in patients with type 2 diabetes [9]. These findings suggest that peripheral blood monocytes may reflect liver fibrosis risk and treatment-related changes in patients with type 2 diabetes and MASLD.

SGLT2, encoded by solute carrier family 5 member 2 (SLC5A2), is predominantly expressed in renal proximal tubular cells, and SLC5A2 is classified as a kidney-enriched gene [10]. Publicly available data from the Human Protein Atlas indicate that SLC5A2/SGLT2 is generally undetectable in monocytes, macrophages, and other major immune-cell populations at the protein level [11]. However, Semo et al. reported SLC5A2 transcripts and cell-surface SGLT2 immunoreactivity detected by flow cytometry in primary human monocytes [12]. SGLT2 mRNA expression in peripheral blood mononuclear cells (PBMCs) has also been associated with inflammatory cytokine levels and blood glucose concentrations in proliferative diabetic retinopathy [13].

We were interested in whether baseline expression of the target gene in a MASLD-relevant cell lineage was associated with changes in liver function during treatment with the corresponding drug class. Therefore, we focused on monocyte SGLT2 mRNA expression as a transcriptomic feature that may characterize interindividual heterogeneity in liver-related biochemical changes during SGLT2 inhibitor treatment. Specifically, this study investigated whether baseline monocyte SGLT2 mRNA expression was associated with changes in the FIB-4 index after 6 months of luseogliflozin treatment in participants with type 2 diabetes and MASLD.

## Materials and methods

### Study design and participants

**Fig. 1:**
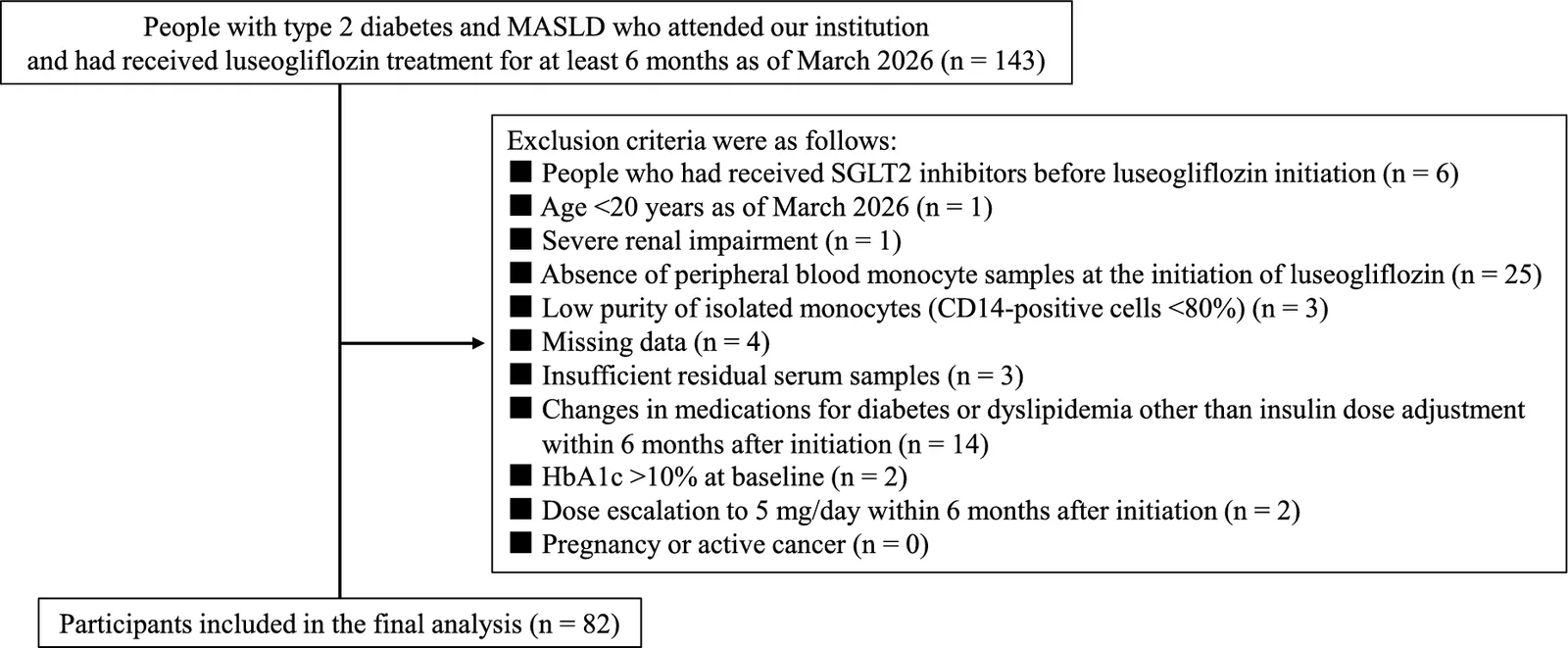
Flowchart showing the participant selection process. The process of selecting study participants is shown. As of March 2026, a total of 143 people with type 2 diabetes and MASLD who had received luseogliflozin treatment for at least 6 months were screened for eligibility. After applying the exclusion criteria, 82 participants were included in the final analysis. HbA1c, hemoglobin A1c; MASLD, metabolic dysfunction-associated steatotic liver disease; SGLT2, sodium-glucose cotransporter 2

This single-center retrospective observational study used prospectively collected peripheral blood monocyte samples from participants with type 2 diabetes and hepatic steatosis corresponding to MASLD, who attended the outpatient Department of Internal Medicine at Nerima Hikarigaoka Hospital between October 2023 and March 2026. MASLD was defined as imaging-confirmed hepatic steatosis together with at least one cardiometabolic risk factor; all included participants met this criterion because they had type 2 diabetes [14]. All regularly followed people with type 2 diabetes and fatty liver disease were screened for eligibility. Because the screened population was restricted to participants with type 2 diabetes, people with type 1 diabetes were not included in the initial study population. Eligible participants had type 2 diabetes and imaging-confirmed hepatic steatosis before luseogliflozin initiation [14]; no prior use of other SGLT2 inhibitors; and at least 6 months of luseogliflozin treatment as of March 2026. Luseogliflozin was initiated and continued at 2.5 mg/day. Participants were excluded for age <20 years as of March 2026; pregnancy; active cancer; renal replacement therapy or estimated glomerular filtration rate (eGFR) <15 mL/min/1.73 m^2^; absence of baseline monocyte samples; low purity of isolated monocytes, defined as CD14-positive cells <80%; missing data; insufficient residual serum; changes in antidiabetic or lipid-lowering medications within 6 months, except insulin dose adjustment; baseline hemoglobin A1c (HbA1c) >10%; temporary interruption of luseogliflozin because of adverse events; or dose escalation to 5 mg/day within 6 months. Peripheral blood samples for monocyte isolation had been prospectively collected at SGLT2 inhibitor initiation from consenting participants with type 2 diabetes under an Institutional Review Board-approved prospective observational protocol. Samples for polymerase chain reaction analysis were processed on the collection day and stored at −80 °C according to the institutional protocol. Participants with baseline HbA1c >10% were excluded because they may require prior insulin therapy for severe hyperglycemia or be at risk of acute metabolic decompensation, making them unsuitable for evaluating luseogliflozin effects. During the study period, 143 people with type 2 diabetes and MASLD had received luseogliflozin treatment for at least 6 months. After applying the inclusion and exclusion criteria, 82 participants were included in the final analysis (Fig. 1). The primary outcome was the change in the FIB-4 index over 6 months. The prospective monocyte collection and analysis and the retrospective analysis of clinical and laboratory data related to luseogliflozin treatment were approved by the Institutional Review Board. All participants provided written informed consent for monocyte collection and analysis. Additional consent for retrospective clinical data analysis was waived by the Institutional Review Board, and study information was disclosed using an opt-out approach.

### Clinical data collection

**Fig. 2:**
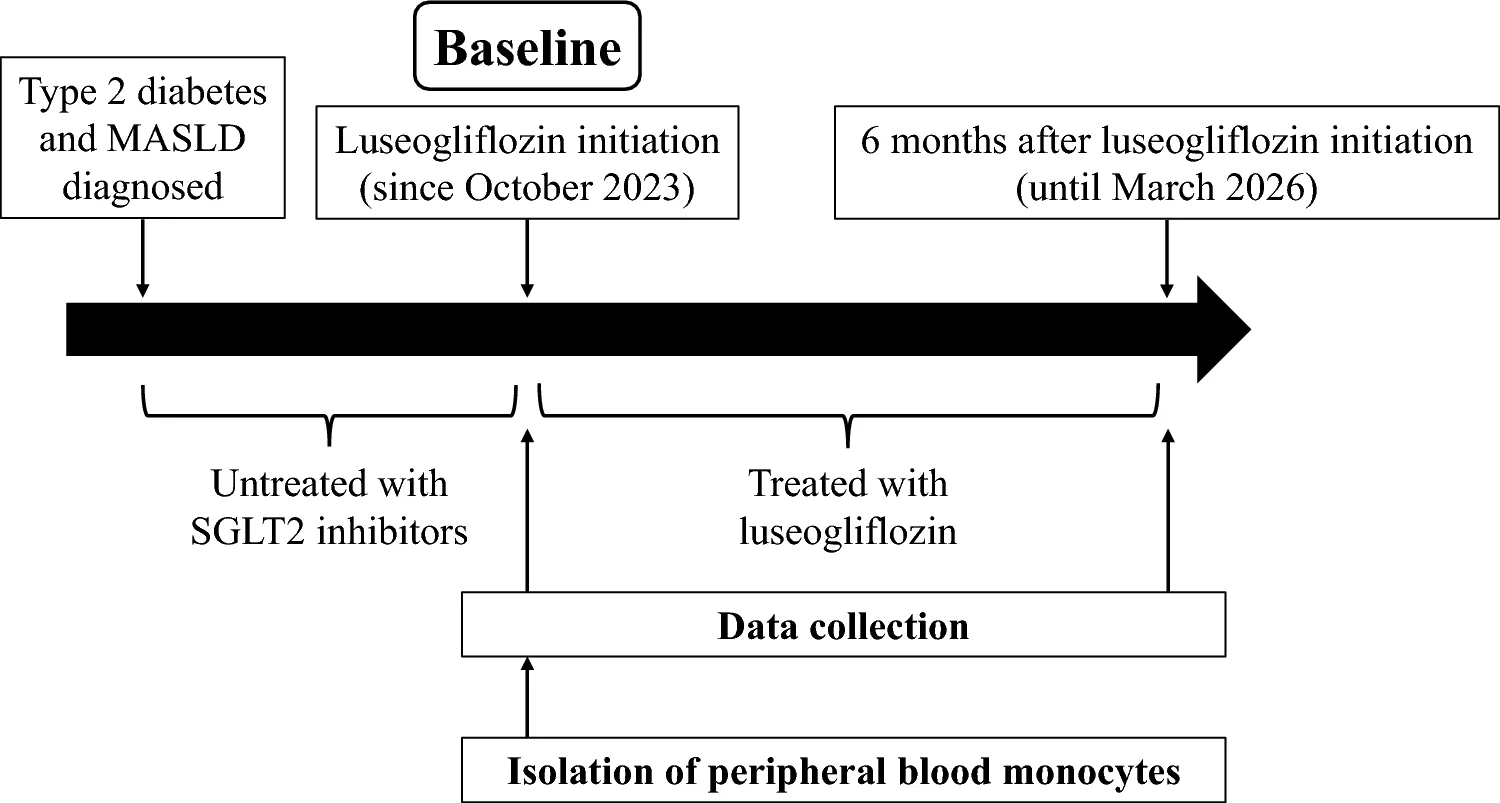
Timeline of this study. Clinical data were collected at baseline, defined as the date of luseogliflozin initiation, and at 6 months after treatment initiation. Peripheral blood monocytes were isolated at baseline only. MASLD, metabolic dysfunction-associated steatotic liver disease; SGLT2, sodium-glucose cotransporter 2

The study timeline is shown in Fig. 2. Peripheral blood monocytes were collected at baseline, defined as luseogliflozin initiation. Clinical and laboratory data were obtained at baseline and 6 months. Demographic and clinical data included age, sex, diabetes duration, smoking status, body weight, body mass index (BMI), and waist circumference. Current smoker was defined as a participant who reported active cigarette smoking at baseline, defined as the time of luseogliflozin initiation, regardless of the number of cigarettes smoked per day. Physiological measurements included systolic blood pressure (SBP) and diastolic blood pressure (DBP). Data on diabetic complications, including neuropathy, retinopathy, nephropathy, and atherosclerotic disease, were also collected. Laboratory parameters measured during routine care included HbA1c, triglycerides (TG), high-density lipoprotein cholesterol (HDL-C), low-density lipoprotein cholesterol (LDL-C), aspartate aminotransferase (AST), alanine aminotransferase (ALT), γ-glutamyl transpeptidase (γ-GTP), uric acid (UA), platelet count, urinary albumin-to-creatinine ratio (UACR), and eGFR. Serum high-sensitivity C-reactive protein (hsCRP) was measured using residual serum samples. Additional peripheral blood for monocyte isolation was collected only at baseline with consent. Biochemical data were available for all participants. Blood samples were obtained in the morning after ≥12 h of fasting. HbA1c was measured using a latex agglutination assay. eGFR was calculated using the Chronic Kidney Disease Epidemiology Collaboration equation modified for Japanese individuals [15]. Urinary albumin and creatinine were measured using turbidimetric immunoassay and enzymatic methods, respectively, and UACR was calculated from spot urine samples. The FIB-4 index was calculated as age (years) × AST (U/L) / [platelet count (× 10^9^/L) × √ALT (U/L)] and extracted from electronic medical records as a non-invasive marker of liver fibrosis in MASLD [16]. FIB-4 values <1.3 were considered low risk, values of 1.3–2.67 were considered intermediate risk, and values ≥2.67 were considered high risk for advanced fibrosis [17]. Serum hsCRP was measured in duplicate using an immunoturbidimetric assay (CRP-Latex; Kamiya Biomedical Company, WA, USA) according to the manufacturer’s protocol. Samples with duplicate values differing by more than 3 standard deviations were reanalyzed. Diabetic nephropathy was defined as UACR ≥30 mg/gCr and/or eGFR <30 mL/min/1.73 m^2^ in the absence of other primary kidney diseases, according to the criteria of the Joint Committee on Diabetic Nephropathy in Japan [18]. Diabetic retinopathy was assessed by ophthalmologists according to the International Clinical Diabetic Retinopathy Disease Severity Scale [19]. Diabetic neuropathy was assessed based on medical records according to the simplified diagnostic criteria proposed by the Diabetic Neuropathy Study Group in Japan [20].

### Isolation of peripheral blood monocytes

Peripheral blood samples were collected in the early morning after an overnight fast into 10-mL K2EDTA Vacutainer tubes. Peripheral blood monocytes were directly isolated from whole blood using the EasySep Direct Human Monocyte Isolation Kit and EasyEights EasySep Magnet (STEMCELL Technologies, Vancouver, Canada), according to the manufacturer’s protocol. This column-free immunomagnetic negative selection method was selected to minimize cell activation during the isolation procedure. The purity of isolated monocytes was assessed by flow cytometry using phycoerythrin-conjugated anti-human CD14 antibody (BioLegend, CA, USA) and a FACSCelesta flow cytometer (Becton, Dickinson and Company, NJ, USA), as previously described [21]. Samples with monocyte purity <80% were excluded. Accordingly, data from three participants were excluded because of low monocyte purity, with CD14-positive cell percentages of 75%, 77%, and 79%. Isolated monocytes were resuspended in RPMI 1640 medium supplemented with 10% fetal bovine serum and 1× antibiotic–antimycotic solution (Thermo Fisher Scientific, Waltham, MA, USA). Cells were seeded in 24-well culture plates (Corning, Tewksbury, MA, USA) at 1 × 10^6^ cells/well and cultured for 24 h at 37 °C in 5% CO_2_. After adhesion was confirmed, the medium was replaced with RPMI 1640 containing 0.25% bovine serum albumin for 2 h of serum starvation. Cultured monocytes were then harvested, and total RNA was extracted immediately after cell collection.

### Quantitative real-time polymerase chain reaction analysis

Total RNA was extracted from cultured human monocytes using TRIzol reagent (Thermo Fisher Scientific), as previously described [22]. Reverse transcription was performed using random primers (Thermo Fisher Scientific) and ReverTra Ace reverse transcriptase (Toyobo, Osaka, Japan). Quantitative real-time polymerase chain reaction (qRT-PCR) was performed using a ViiA 7 Real-Time PCR System and Fast SYBR Green Master Mix (Thermo Fisher Scientific), according to the manufacturer’s protocol. The mRNA expression levels of SGLT2 were normalized to those of β-actin, and relative quantification was performed using the comparative threshold cycle method. Monocyte-derived gene expression data were available for all participants. Primer sequences are provided in Supplementary Table S1. Ct values for SGLT2 and β-actin are summarized in Supplementary Table S2.

### Outcome measures

The primary outcome was the change in the FIB-4 index from baseline to 6 months after luseogliflozin initiation. The change in the FIB-4 index was calculated as the value at 6 months minus the baseline value; therefore, a negative value indicated a reduction in the FIB-4 index.

### Statistical analyses

Statistical analyses were performed using Prism 9 (GraphPad Software, San Diego, CA, USA). Continuous variables with a normal distribution were analyzed using Student’s t-test, whereas non-normally distributed variables were analyzed using the Mann–Whitney U test or Wilcoxon signed-rank test, as appropriate. Categorical variables were analyzed using the chi-square test or Fisher’s exact test. Associations between variables were assessed using Spearman’s rank correlation coefficient. Multiple regression analysis was performed using the change in the FIB-4 index at 6 months as the dependent variable. Model 1 was constructed using a stepwise selection method. Model 2 was constructed by adding baseline waist circumference and HbA1c as forced-entry covariates to the variables selected in Model 1. HbA1c and waist circumference were selected from a previous clinical study showing that luseogliflozin significantly reduced HbA1c and waist circumference, along with improvements in hepatic function and liver-related indices, in patients with type 2 diabetes complicated by hepatic dysfunction [23]. Additionally, Model 3 was constructed by adding changes in waist circumference, HbA1c, and ALT as forced-entry covariates to the variables selected in Model 1. Because ALT is a mathematical component of the FIB-4 index, Model 3 was regarded as an exploratory sensitivity analysis. Furthermore, additional exploratory sensitivity analyses were constructed by adding baseline duration of diabetes, waist circumference, HbA1c, eGFR, and hsCRP as forced-entry covariates to the variables selected in Model 1. The sensitivity analyses were conducted using the change in the FIB-4 index at 6 months or the FIB-4 index at the 6-month mark as the dependent variable. Non-normally distributed continuous variables, including baseline monocyte SGLT2 mRNA expression, were log-transformed before being entered into regression models. Model fit was evaluated using the adjusted coefficient of determination (adjusted *R*^2^) and Akaike information criterion (AIC). Lower AIC values indicated better relative fit among models constructed using the same dependent variable and analytical dataset. All P values were two-sided, and *P* < 0.05 was considered statistically significant.

## Results

The baseline characteristics of the study population are presented in Table 1. To examine differences in clinical characteristics according to baseline monocyte SGLT2 mRNA expression, participants were divided into low- and high-expression groups according to the median expression level, with 41 participants in each group. Compared with the low-expression group, the high-expression group was younger and had a shorter duration of diabetes, a lower platelet count, a higher ALT level, and a higher baseline FIB-4 index. No significant between-group differences were observed in the other clinical characteristics, diabetic complications, physiological measurements, most laboratory parameters, or medication use.

**Table 1:** Baseline characteristics according to baseline monocyte SGLT2 mRNA expression levels

	Overall (*n* = 82)	Low SGLT2 expression (*n* = 41)	High SGLT2 expression (*n* = 41)	*P-*value
*Clinical characteristics*				
Age (years)	53.8±10.2	56.2±9.3	51.4±10.7	0.035
Female sex, *n* (%)	42 (51)	23 (56)	19 (46)	0.508
Duration of diabetes (years)	11 (4–20)	13 (7–21)	6 (2–14)	0.010
Current Smoker, *n* (%)	26 (32)	12 (29)	14 (34)	0.813
Body weight (kg)	69.0±11.9	68.6±11.8	69.3±12.1	0.777
BMI (kg/m^2^)	25.7±3.2	25.4±2.8	25.9±3.5	0.465
Waist circumference (cm)	88.4±7.6	87.0±4.5	89.8±9.7	0.109
*Complications of diabetes, n (%)*				
Neuropathy	42 (51)	21 (51)	21 (51)	1.000
Retinopathy	31 (38)	16 (39)	15 (37)	1.000
Nephropathy	46 (56)	24 (59)	22 (54)	0.824
Atherosclerotic diseases	24 (29)	15 (37)	9 (22)	0.225
*Physiological testing*				
SBP (mmHg)	130±9	130±9	130±9	0.747
DBP (mmHg)	69±6	69±6	70±6	0.373
*Laboratory parameters*				
HbA1c (%)	8.0 (7.4–8.9)	7.9 (7.4–8.9)	8.3 (7.4–8.9)	0.871
TG (mg/dL)	240 (165–319)	237 (174–294)	292 (158–324)	0.504
HDL-C (mg/dL)	43 (37–47)	43 (37–47)	42 (39–47)	0.974
LDL-C (mg/dL)	109 (95–119)	106 (93–118)	110 (101–119)	0.224
AST (IU/L)	28 (18–34)	25 (16–32)	29 (24–36)	0.076
ALT (IU/L)	38 (28–44)	36 (27–40)	41 (30–45)	0.020
γ-GTP (IU/L)	60 (45–70)	55 (46–70)	62 (34–70)	0.735
UA (mg/dL)	5.6 (4.1–6.8)	5.5 (4.1–6.8)	5.8 (4.2–6.7)	0.484
Platelet count (× 10^9^/L)	230 (170–260)	250 (220–260)	220 (150–250)	0.012
UACR (mg/gCr)	33.3 (10.1–54.2)	34.9 (11.2–54.5)	33.0 (8.7–50.0)	0.280
eGFR (mL/min/1.73m^2^)	63.9±12.2	66.3±11.9	61.6±12.2	0.081
hsCRP (mg/L)	2.9±0.8	2.8±0.7	3.0±0.8	0.197
FIB-4 index	1.17±0.52	1.04±0.35	1.30±0.62	0.024
SGLT2 mRNA expression in monocytes (× 10^–2^)	4.36 (0.47–20.95)	0.46 (0.10–1.28)	21.00 (12.24–31.49)	<0.001
*Medications, n (%)*				
Insulin	17 (21)	7 (17)	10 (24)	0.587
Sulfonylureas	8 (10)	5 (12)	3 (7)	0.712
Glinides	8 (10)	4 (10)	4 (10)	1.000
GLP-1RAs	35 (43)	18 (44)	17 (41)	1.000
DPP4 inhibitors	26 (32)	13 (32)	13 (32)	1.000
Metformin	53 (65)	25 (61)	28 (68)	0.645
TZDs	7 (9)	5 (12)	2 (5)	0.432
Alpha-GIs	5 (6)	4 (10)	1 (2)	0.359
ARBs	49 (60)	25 (61)	24 (59)	1.000
CCBs	13 (16)	7 (17)	6 (15)	1.000
Statins	59 (72)	30 (73)	29 (71)	1.000
Fibrates	36 (44)	21 (51)	15 (37)	0.266
UA lowering agents	20 (24)	11 (27)	9 (22)	0.798

Participants were divided into low and high monocyte SGLT2 expression groups according to the median baseline monocyte SGLT2 mRNA expression level. Data are expressed as the mean ± standard deviation, median (interquartile range), or number (%). Current smoker was defined as active cigarette smoking at baseline, regardless of the number of cigarettes smoked per day. ALT, alanine aminotransferase; ARBs, angiotensin receptor blockers; AST, aspartate aminotransferase; BMI, body mass index; CCBs, calcium channel blockers; DBP, diastolic blood pressure; DPP4, dipeptidyl peptidase−4; eGFR, estimated glomerular filtration rate; FIB-4, fibrosis-4; γ-GTP, γ-glutamyl transpeptidase; GIs, glycosidase inhibitors; GLP-1RAs, glucagon-like peptide−1 receptor agonists; HbA1c, hemoglobin A1c; HDL-C, high-density lipoprotein cholesterol; hsCRP, high-sensitivity C-reactive protein; LDL-C, low-density lipoprotein cholesterol; SBP, systolic blood pressure; SGLT2, sodium-glucose cotransporter 2; TG, triglycerides; TZDs, thiazolidinediones; UA, uric acid; UACR, urine albumin-to-creatinine ratio

Spearman’s rank correlation analysis was performed to assess the associations between monocyte SGLT2 mRNA expression and baseline clinical parameters (Supplementary Table S3). Monocyte SGLT2 mRNA expression was negatively correlated with age and duration of diabetes, but not with most metabolic, renal, inflammatory, or liver-related parameters.

We next evaluated changes in clinical parameters during 6 months of luseogliflozin treatment (Table 2). Waist circumference, SBP, HbA1c, AST, ALT, UACR, hsCRP, and the FIB-4 index decreased significantly from baseline to 6 months, whereas HDL-C and platelet counts increased significantly. The FIB-4 index decreased from 1.17 ± 0.52 to 0.87 ± 0.41 (*P* < 0.001). Body weight, BMI, DBP, TG, LDL-C, γ-GTP, UA, and eGFR did not change significantly.

**Table 2:** Changes in clinical parameters from baseline to 6 months after initiation of luseogliflozin

	Overall (*n* = 82)		
	Baseline	After	*P-*value
Body weight (kg)	69.0±11.9	68.9±12.0	0.883
BMI (kg/m^2^)	25.7±3.2	25.7±3.3	0.942
Waist circumference (cm)	88.4±7.6	87.3±6.9	0.003
SBP (mmHg)	130±9	127±8	0.011
DBP (mmHg)	69±6	68±6	0.273
HbA1c (%)	8.0 (7.4–8.9)	7.3 (6.6–8.6)	<0.001
TG (mg/dL)	240 (165–319)	196 (150–315)	0.664
HDL-C (mg/dL)	43 (37–47)	47 (40–51)	<0.001
LDL-C (mg/dL)	109 (95–119)	108 (96–121)	0.923
AST (IU/L)	28 (18–34)	26 (18–30)	<0.001
ALT (IU/L)	38 (28–44)	29 (19–38)	<0.001
γ-GTP (IU/L)	60 (45–70)	58 (42–73)	0.458
UA (mg/dL)	5.6 (4.1–6.8)	5.5 (3.2–6.9)	0.356
Platelet count (× 10^9^/L)	230 (170–260)	285 (243–348)	<0.001
UACR (mg/gCr)	33.3 (10.1–54.2)	20.9 (8.8–38.4)	<0.001
eGFR (mL/min/1.73m^2^)	63.9±12.2	63.8±12.9	0.907
hsCRP (mg/L)	2.9±0.8	2.1±1.0	<0.001
FIB-4 index	1.17±0.52	0.87±0.41	<0.001

Data are expressed as the mean ± standard deviation, or the median (interquartile range), and are compared using the paired *t*-test or the Wilcoxon signed-rank test. ALT, alanine aminotransferase; AST, aspartate aminotransferase; BMI, body mass index; DBP, diastolic blood pressure; eGFR, estimated glomerular filtration rate; FIB-4, fibrosis-4; γ-GTP, γ-glutamyl transpeptidase; HbA1c, hemoglobin A1c; HDL-C, high-density lipoprotein cholesterol; hsCRP, high-sensitivity C-reactive protein; LDL-C, low-density lipoprotein cholesterol; SBP, systolic blood pressure; TG, triglycerides; UA, uric acid; UACR, urine albumin-to-creatinine ratio

Because the FIB-4 index decreased after treatment, we investigated whether baseline monocyte SGLT2 mRNA expression was associated with changes in clinical parameters using Spearman’s rank correlation analysis (Table 3). Baseline monocyte SGLT2 mRNA expression was negatively correlated with changes in waist circumference (ρ = −0.269, *P* = 0.015), AST (ρ = −0.264, *P* = 0.017), ALT (ρ = −0.245, *P* = 0.027), platelet count (ρ = 0.431, *P* < 0.001), hsCRP (ρ = −0.239, *P *= 0.031), and FIB-4 index (ρ = −0.501, *P* < 0.001). Baseline monocyte SGLT2 mRNA expression was not significantly correlated with the change in HbA1c (ρ = 0.056, *P* = 0.620). Similarly, no significant correlations were observed with changes in body weight, BMI, blood pressure, lipid parameters, UA, UACR, or eGFR.

**Table 3:** Correlation analysis between baseline monocyte SGLT2 mRNA expression and changes in clinical parameters

	SGLT2 mRNA expression in monocytes	
	*ρ*	*P*-value
ΔBody weight (kg)	0.084	0.452
ΔBMI (kg/m^2^)	0.080	0.476
ΔWaist circumference (cm)	−0.269	0.015
ΔSBP (mmHg)	−0.002	0.984
ΔDBP (mmHg)	−0.118	0.289
ΔHbA1c (%)	0.056	0.620
ΔTG (mg/dL)	−0.123	0.270
ΔHDL-C (mg/dL)	0.203	0.067
ΔLDL-C (mg/dL)	−0.091	0.419
ΔAST (IU/L)	−0.264	0.017
ΔALT (IU/L)	−0.245	0.027
Δγ-GTP (IU/L)	−0.142	0.204
ΔUA (mg/dL)	0.102	0.362
ΔPlatelet count (× 10^9^/L)	0.431	<0.001
ΔUACR (mg/gCr)	−0.095	0.399
ΔeGFR (mL/min/1.73m^2^)	0.165	0.139
ΔhsCRP (mg/L)	−0.239	0.031
ΔFIB-4 index	−0.501	<0.001

Spearman’s rank correlation analysis was performed. The change in each parameter (Δ) was calculated by subtracting the baseline value from the value obtained at 6 months after the initiation of luseogliflozin. ALT, alanine aminotransferase; AST, aspartate aminotransferase; BMI, body mass index; DBP, diastolic blood pressure; eGFR, estimated glomerular filtration rate; FIB-4, fibrosis-4; γ-GTP, γ-glutamyl transpeptidase; HbA1c, hemoglobin A1c; HDL-C, high-density lipoprotein cholesterol; hsCRP, high-sensitivity C-reactive protein; LDL-C, low-density lipoprotein cholesterol; SBP, systolic blood pressure; SGLT2, sodium-glucose cotransporter 2; *ρ,* Spearman's correlation coefficient; TG, triglycerides; UA, uric acid; UACR, urine albumin-to-creatinine ratio

Finally, to determine whether baseline monocyte SGLT2 mRNA expression was independently associated with change in the FIB-4 index, simple and multiple linear regression analyses were performed with change in the FIB-4 index at 6 months as the dependent variable. In simple linear regression analyses of baseline clinical parameters, waist circumference (β = −0.023, *P* = 0.003), AST (β = −0.450, *P* = 0.003), eGFR (β = 0.011, *P* = 0.022), platelet counts (β = 1.393,* P* < 0.001), hsCRP (β = −0.177, *P* = 0.028), baseline FIB-4 index (β = −0.760, *P* < 0.001), and monocyte SGLT2 mRNA expression (β = −0.122, *P* < 0.001) were significantly associated with change in the FIB-4 index (Supplementary Table S4). In the multiple regression analysis, Model 1 included variables selected using a stepwise procedure (age and baseline FIB-4 index), and monocyte SGLT2 mRNA expression remained significantly associated with change in the FIB-4 index (β = −0.066, *P* < 0.001; Table 4). In Model 2, additional adjustment was made for baseline waist circumference and HbA1c, together with the variables included in Model 1, and monocyte SGLT2 mRNA expression remained significantly associated with change in the FIB-4 index (β = −0.064, *P* < 0.001; Table 4). Additionally, in Model 3, additional adjustment was made for the changes in waist circumference, HbA1c, and ALT, together with the variables included in Model 1, and monocyte SGLT2 mRNA expression remained significantly associated with change in the FIB-4 index (β = −0.064, *P* < 0.001; Table 4). Furthermore, in two additional exploratory sensitivity analyses, baseline duration of diabetes, waist circumference, HbA1c, eGFR, and hsCRP were added to the variables included in Model 1. Baseline monocyte SGLT2 mRNA expression remained significantly associated both with the change in the FIB-4 index (β = −0.068, *P* < 0.001; Supplementary Table S5) and with the FIB-4 index at 6 months (β = −0.068, *P* < 0.001; Supplementary Table S6) after full adjustment. These findings support an association between baseline monocyte SGLT2 mRNA expression and the magnitude of FIB-4 index reduction during luseogliflozin treatment.

**Table 4:** Multiple linear regression models examining the association between baseline monocyte SGLT2 mRNA expression and change in the FIB-4 index after 6 months of luseogliflozin treatment

	Unstandardized β	95% CI	*P*-value
Dependent variable: Change in the FIB-4 index			
Simple regression model	(Adjusted *R*^2^ = 0.296, AIC = − 123.39)		
SGLT2 mRNA expression in monocytes ^†^	−0.122	−0.163 to − 0.081	<0.001
Age- and sex-adjusted model	(Adjusted *R*^2^ = 0.350, AIC = − 128.02)		
SGLT2 mRNA expression in monocytes ^†^	−0.133	−0.174 to − 0.091	<0.001
Age	−0.005	−0.015–0.005	0.339
Female sex	−0.277	−0.475 to − 0.079	0.007
Multiple regression model 1	(Adjusted *R*^2^ = 0.670, AIC = − 183.72)		
SGLT2 mRNA expression in monocytes ^†^	−0.066	−0.098 to − 0.034	<0.001
Age	0.012	0.004–0.020	0.005
FIB-4 index	−0.748	-0.904 to − 0.592	<0.001
Multiple regression model 2	(Adjusted *R*^2^ = 0.669, AIC = − 181.41)		
SGLT2 mRNA expression in monocytes ^†^	−0.064	−0.096 to − 0.032	<0.001
Age	0.012	0.003–0.020	0.008
FIB-4 index	−0.721	−0.885 to − 0.557	<0.001
Waist circumference	−0.004	−0.014–0.006	0.381
HbA1c ^†^	−0.287	− 0.936 to 0.361	0.380
Multiple regression model 3	(Adjusted *R*^2^ = 0.668, AIC = − 180.34)		
SGLT2 mRNA expression in monocytes ^†^	−0.064	−0.098 to − 0.030	<0.001
Age	0.012	0.004–0.021	0.005
FIB-4 index	−0.755	−0.924 to − 0.587	<0.001
ΔWaist circumference	0.011	−0.013 to 0.036	0.360
ΔHbA1c	−0.031	−0.113 to 0.051	0.452
ΔALT	−0.003	−0.012 to 0.007	0.549

A multiple linear regression analysis with crude and adjusted beta coefficients. Independent variables that were not measures of change corresponded to the values obtained at the baseline. The change in each parameter (Δ) was calculated by subtracting the baseline value from the value obtained at 6 months after the initiation of luseogliflozin. The change in FIB-4 index was calculated as the difference between values at 6 months and baseline (FIB-4 index at 6 months − baseline FIB-4 index), with negative values indicating a reduction in the FIB-4 index. Adjusted *R*^2^, adjusted coefficient of determination; AIC, Akaike information criterion; β, partial regression coefficient; CI, confidence interval; FIB-4, fibrosis-4; HbA1c, hemoglobin A1c; SGLT2, sodium-glucose cotransporter 2. ^†^ Log-transformed using the natural logarithm

## Discussion

In the present study, 6 months of luseogliflozin treatment was associated with significant reductions in waist circumference, HbA1c, liver enzyme levels, hsCRP, UACR, and the FIB-4 index in participants with type 2 diabetes and MASLD, whereas body weight did not change significantly. The principal finding was that higher baseline SGLT2 mRNA expression in peripheral blood monocytes was associated with a greater reduction in the FIB-4 index. This association remained significant after multivariable adjustment and in additional sensitivity analyses accounting for baseline FIB-4 and other clinical variables.

The present analysis was guided by both pharmacological and pathophysiological considerations: SGLT2 is the canonical molecular target of luseogliflozin, whereas the monocyte–macrophage lineage contributes to MASLD-associated inflammation and fibrogenesis. Accordingly, monocyte SGLT2 mRNA may represent a pharmacologically anchored, hypothesis-generating transcriptomic feature associated with subsequent changes in the FIB-4 index during luseogliflozin treatment. Although the present study does not establish functional SGLT2 protein expression, direct target engagement in monocytes, or treatment specificity, the observed association provides a rationale for future mechanistic studies and for evaluating the potential value of monocyte SGLT2 mRNA in treatment stratification.

The observed hepatic and metabolic changes are broadly consistent with previous studies suggesting favorable effects of SGLT2 inhibitors on hepatic steatosis and fibrosis-related indices in MASLD [4, 24]. Luseogliflozin has also been reported to improve liver enzymes, fatty liver index, and hepatic fat accumulation in patients with type 2 diabetes and MASLD [5, 6, 25]. In the present cohort, waist circumference decreased despite no significant change in body weight, suggesting that body weight alone may not fully capture changes in abdominal adiposity or fat distribution during SGLT2 inhibitor treatment; a similar dissociation between hepatic improvement and body weight reduction has been reported with ipragliflozin [26]. Nevertheless, the reduction in the FIB-4 index requires cautious interpretation because the index is calculated from age, AST, ALT, and platelet count [2, 16, 17]. Because AST and ALT decreased and platelet count increased, the observed change likely reflects changes in these laboratory components and should not be interpreted as evidence of histological fibrosis regression.

The biological rationale for focusing on monocytes is supported by the involvement of the monocyte–macrophage lineage in MASLD progression. Single-cell and spatial transcriptomic studies have identified heterogeneous hepatic macrophage populations, including monocyte-derived, lipid-associated, TREM2-positive, and scar-associated macrophages, near sites of hepatocellular injury and fibrosis [27–29]. Analysis of isolated monocytes may therefore provide a more cell-resolved signal than analysis of total PBMCs, although the present study did not directly compare these cellular compartments [30]. The inverse associations of monocyte SGLT2 mRNA expression with age and diabetes duration may also suggest a relationship with diabetes-associated immunometabolic changes. Chronic hyperglycemia can induce persistent metabolic and epigenetic reprogramming of monocytes and macrophages [31, 32], and previous studies have associated SGLT2 mRNA expression in PBMCs with inflammatory cytokines and glycemia [13]. SGLT2 inhibitors may also modify immune-cell function indirectly through systemic metabolic changes, as illustrated by the reported suppression of macrophage NLRP3 inflammasome activation during empagliflozin treatment [33]. Taken together, baseline monocyte SGLT2 mRNA may reflect interindividual differences in monocyte immunometabolic state rather than direct inhibition of monocyte SGLT2 by luseogliflozin.

The RNA-level measurement should be interpreted cautiously. Publicly available data indicate that SLC5A2 is kidney-enriched and that SGLT2 protein is generally undetectable in monocytes [10, 11], whereas Semo et al. reported low-level SLC5A2 transcripts and cell-surface SGLT2 immunoreactivity in primary human monocytes [12]. However, knockout-based characterization has demonstrated substantial differences in the specificity of commercially available anti-SGLT2 antibodies, making low-level extra-renal immunoreactivity difficult to interpret [34]. Thus, measurable SGLT2 mRNA does not establish corresponding protein abundance, transporter activity, or direct SGLT2-dependent signaling. Moreover, because relative expression was calculated using the comparative Ct method, differences in normalized Ct values are expressed on an exponential scale and may therefore result in large fold differences in relative expression. Accordingly, the wide interindividual range in relative SGLT2 mRNA expression should not be interpreted as an equivalent difference in absolute transcript or protein abundance, particularly because SGLT2 protein expression was not assessed. Because the relative expression values were non-normally distributed, they were natural-log transformed in the regression analyses, and summary statistics, ranges, and detection rates for the SGLT2 and β-actin Ct values are provided to improve measurement transparency.

The group with high SGLT2 mRNA expression was younger and had a shorter duration of diabetes, a lower platelet count, and a higher baseline FIB-4 index, whereas most other baseline clinical and laboratory parameters did not differ significantly between the groups. Because platelet count is a component of the FIB-4 formula, this difference may have partly contributed to the higher baseline FIB-4 index and raised the possibility that the greater subsequent reduction reflected baseline imbalance or regression to the mean. However, baseline monocyte SGLT2 mRNA remained significantly associated with the 6-month FIB-4 index after adjustment for baseline FIB-4 and other clinical variables, making it less likely that the association was explained solely by baseline differences. Nevertheless, residual confounding and regression to the mean cannot be completely excluded. In addition, the single-center design, selected outpatient population, fixed luseogliflozin dose, and exclusion of participants with medication changes or incomplete data limit generalizability. Monocyte SGLT2 mRNA measurement currently has no established clinical utility: this study did not determine a clinically applicable cutoff; evaluate discrimination, calibration, or incremental predictive performance; or perform external validation. The current ex vivo protocol is also unsuitable for routine clinical implementation.

Several limitations should be acknowledged. First, this was a single-center retrospective observational study without a non-SGLT2 inhibitor control group or an external validation cohort. Therefore, causal relationships, treatment specificity, and generalizability cannot be established. The study also lacked comparator groups without type 2 diabetes or MASLD, preventing assessment of whether monocyte SGLT2 mRNA expression differs according to these conditions. Second, the outcome was limited to the FIB-4 index. Liver stiffness measurement by vibration-controlled transient elastography or magnetic resonance elastography, serum fibrosis markers such as the Enhanced Liver Fibrosis test or Mac-2 binding protein glycosylation isomer, and histological assessment were unavailable; therefore, the observed change could not be corroborated using independent fibrosis assessments. Body composition, visceral fat area, skeletal muscle mass, and hepatic fat content were also not directly assessed. Third, monocyte SGLT2 mRNA was measured only at baseline, and SGLT2 protein expression, transporter activity, and monocyte functional responses were not assessed. The study also did not compare isolated monocytes with total PBMCs or other immune-cell subsets. Furthermore, mRNA expression was measured after 24 h of culture followed by 2 h of serum starvation rather than immediately after isolation. Although all samples were processed using the same protocol, these procedures may have altered monocyte transcriptional state; consequently, the measured values may reflect both interindividual biological differences and responses to the standardized ex vivo conditions.

In conclusion, higher baseline SGLT2 mRNA expression in peripheral blood monocytes was associated with a greater reduction in the FIB-4 index after 6 months of luseogliflozin treatment in participants with type 2 diabetes and MASLD in this single-center cohort. Monocyte SGLT2 mRNA expression may represent an exploratory, hypothesis-generating, cell-specific transcriptomic feature associated with interindividual variation in FIB-4 index changes, rather than evidence of functional SGLT2 signaling in monocytes or histological fibrosis regression. These findings may provide a foundation for future mechanistic and treatment-stratification studies; however, their biological and clinical significance requires validation in independent cohorts using direct protein and functional analyses and validated methods for fibrosis assessment.

## Supplementary Information

Below is the link to the electronic supplementary material.

Supplementary file1 (DOCX 74 KB)

## Data Availability

The datasets analyzed during the current study are not publicly available because they contain potentially identifiable clinical information, but are available from the corresponding author on reasonable request and with appropriate institutional approval.
