# Supplementary material for: Baseline monocyte SGLT2 mRNA expression and changes in the fibrosis-4 index after luseogliflozin treatment in participants with type 2 diabetes and MASLD
Source: Diabetol Int. 2026 Oct 31;18(1):2. doi: 10.1007/s13340-026-00941-2 (PMC13723643; doi:10.1007/s13340-026-00941-2)
Supplement: Supplementary file 1 [file 13340_2026_941_MOESM1_ESM.docx]

**SUPPLEMENTARY MATERIALS**

**Table S1. qRT-PCR primer sequences used in the study**

| Gene | Forward primer sequence  (5' -3') | Reverse primer sequence  (5' -3') |
| --- | --- | --- |
| SGLT2 | ATAAACAGCTGGGCTGTCCC | CGTAACCCATGAGGATGCAG |
| β-actin | GGACTTCGAGCAAGAGATGG | AGCACTGTGTTGGCGTACAG |

qRT-PCR, quantitative real-time polymerase chain reaction; SGLT2, sodium-glucose cotransporter 2.

**Table S2. Ct values for SGLT2 and β-actin genes in peripheral blood monocytes**

| Gene | Ct value | Detection rate |
| --- | --- | --- |
| SGLT2 | 25.914 (24.103–28.958) | 82/82 (100%) |
| β-actin | 21.702 (21.189–22.125) | 82/82 (100%) |

Ct values are expressed as the median (interquartile range). SGLT2, sodium-glucose cotransporter 2.

**Table S3. Correlation analysis between monocyte SGLT2 mRNA expression and clinical parameters at baseline**

|  | SGLT2 mRNA expression in monocytes | |
| --- | --- | --- |
|  | *ρ* | *P*-value |
| Age (years) | -0.329 | 0.003 |
| Duration of diabetes (years) | -0.451 | <0.001 |
| Body weight (kg) | 0.095 | 0.395 |
| BMI (kg/m^2^) | 0.141 | 0.206 |
| Waist circumference (cm) | 0.163 | 0.143 |
| SBP (mmHg) | 0.029 | 0.794 |
| DBP (mmHg) | 0.125 | 0.264 |
| HbA1c (%) | 0.042 | 0.708 |
| TG (mg/dL) | 0.025 | 0.826 |
| HDL-C (mg/dL) | -0.019 | 0.865 |
| LDL-C (mg/dL) | 0.135 | 0.226 |
| AST (IU/L) | 0.170 | 0.126 |
| ALT (IU/L) | 0.207 | 0.063 |
| γ-GTP (IU/L) | -0.136 | 0.222 |
| UA (mg/dL) | -0.007 | 0.950 |
| Platelet count (×10^9^/L) | -0.219 | 0.071 |
| UACR (mg/gCr) | -0.143 | 0.200 |
| eGFR (mL/min/1.73m^2^) | -0.122 | 0.275 |
| hsCRP (mg/L) | 0.094 | 0.404 |
| FIB-4 index | 0.209 | 0.059 |

Spearman’s rank correlation analysis was performed. All parameters corresponded to the values obtained at baseline. ALT, alanine aminotransferase; AST, aspartate aminotransferase; BMI, body mass index; DBP, diastolic blood pressure; eGFR, estimated glomerular filtration rate; FIB-4, fibrosis-4; γ-GTP, γ-glutamyl transpeptidase; HbA1c, hemoglobin A1c; HDL-C, high-density lipoprotein cholesterol; hsCRP, high-sensitivity C-reactive protein; LDL-C, low-density lipoprotein cholesterol; SBP, systolic blood pressure; SGLT2, sodium-glucose cotransporter 2; *ρ,* Spearman's correlation coefficient; TG, triglycerides; UA, uric acid; UACR, urine albumin-to-creatinine ratio.

**Table S4. Simple linear regression models examining the association between baseline clinical parameters and change in the FIB-4 index after 6 months of luseogliflozin treatment**

| Dependent variable: Change in the FIB-4 index | | | |
| --- | --- | --- | --- |
| Independent variables | Unstandardized β | 95% CI | *P*-value |
| Clinical characteristics |  |  |  |
| Age | 0.005 | -0.007 to 0.017 | 0.439 |
| Female sex | -0.216 | -0.457 to 0.024 | 0.077 |
| Duration of diabetes ^†^ | 0.068 | -0.050 to 0.185 | 0.257 |
| Current Smoker | 0.008 | -0.256 to 0.272 | 0.953 |
| Body weight | -0.001 | -0.011 to 0.010 | 0.946 |
| BMI | -0.008 | -0.047 to 0.030 | 0.666 |
| Waist circumference | -0.023 | -0.039 to -0.008 | 0.003 |
| Complications of diabetes |  |  |  |
| Neuropathy | 0.137 | -0.107 to 0.380 | 0.267 |
| Retinopathy | -0.036 | -0.288 to 0.217 | 0.780 |
| Nephropathy | 0.049 | -0.198 to 0.296 | 0.694 |
| Atherosclerotic diseases | 0.199 | -0.067 to 0.465 | 0.141 |
| Physiological testing |  |  |  |
| SBP | -0.003 | -0.016 to 0.011 | 0.712 |
| DBP | 0.004 | -0.018 to 0.025 | 0.744 |
| Laboratory parameters |  |  |  |
| HbA1c ^†^ | -0.930 | -1.998 to 0.137 | 0.087 |
| TG ^†^ | -0.021 | -0.278 to 0.236 | 0.870 |
| HDL-C ^†^ | 0.342 | -0.419 to 1.103 | 0.374 |
| LDL-C ^†^ | -0.314 | -1.239 to 0.612 | 0.502 |
| AST ^†^ | -0.450 | -0.744 to -0.156 | 0.003 |
| ALT ^†^ | -0.218 | -0.613 to 0.178 | 0.277 |
| γ-GTP ^†^ | -0.013 | -0.284 to 0.257 | 0.923 |
| UA ^†^ | -0.072 | -0.441 to 0.297 | 0.700 |
| Platelet count ^†^ | 1.393 | 1.058 to 1.727 | <0.001 |
| UACR ^†^ | 0.002 | -0.112 to 0.117 | 0.967 |
| eGFR | 0.011 | 0.002 to 0.021 | 0.022 |
| hsCRP | -0.177 | -0.335 to -0.019 | 0.028 |
| FIB-4 index | -0.760 | -0.927 to -0.593 | <0.001 |
| Data regarding monocytes |  |  |  |
| SGLT2 mRNA expression in monocytes ^†^ | -0.122 | -0.163 to -0.081 | <0.001 |
| Medications |  |  |  |
| Insulin | 0.095 | -0.207 to 0.397 | 0.533 |
| Sulfonylureas | 0.036 | -0.378 to 0.449 | 0.863 |
| Glinides | 0.017 | -0.397 to 0.430 | 0.936 |
| GLP-1RAs | 0.075 | -0.173 to 0.322 | 0.551 |
| DPP4 inhibitors | -0.169 | -0.430 to 0.092 | 0.200 |
| Metformin | -0.114 | -0.370 to 0.141 | 0.375 |
| TZDs | -0.039 | -0.478 to 0.400 | 0.859 |
| Alpha-GIs | 0.037 | -0.476 to 0.549 | 0.888 |
| ARBs | 0.013 | -0.237 to 0.263 | 0.918 |
| CCBs | 0.009 | -0.326 to 0.345 | 0.956 |
| Statins | 0.009 | -0.264 to 0.282 | 0.948 |
| Fibrates | -0.053 | -0.300 to 0.194 | 0.670 |
| UA lowering agents | -0.025 | -0.311 to 0.260 | 0.861 |

All independent variables corresponded to the values obtained at baseline. The change in FIB-4 index was calculated as the difference between values at 6 months and baseline (FIB-4 index at 6 months − baseline FIB-4 index), with negative values indicating a reduction in the FIB-4 index. ALT, alanine aminotransferase; ARBs, angiotensin receptor blockers; AST, aspartate aminotransferase; BMI, body mass index; CCBs, calcium channel blockers; DBP, diastolic blood pressure; eGFR, estimated glomerular filtration rate; FIB-4, fibrosis-4; γ-GTP, γ-glutamyl transpeptidase; GIs, glycosidase inhibitors; GLP-1RAs, glucagon-like peptide-1 receptor agonists; HbA1c, hemoglobin A1c; HDL-C, high-density lipoprotein cholesterol; hsCRP, high-sensitivity C-reactive protein; LDL-C, low-density lipoprotein cholesterol; SBP, systolic blood pressure; SGLT2, sodium-glucose cotransporter 2; TG, triglycerides; TZDs, thiazolidinediones; UA, uric acid; UACR, urine albumin-to-creatinine ratio. ^†^ Log-transformed using the natural logarithm.

**Table S5. Exploratory sensitivity analysis using a fully adjusted multiple linear regression model examining the association between baseline monocyte SGLT2 mRNA expression and change in the FIB-4 index after 6 months of luseogliflozin treatment**

| Dependent variable: Change in the FIB-4 index | | | |
| --- | --- | --- | --- |
|  | Unstandardized β | 95% CI | *P*-value |
| Exploratory sensitivity analysis | (Adjusted *R*^2^ = 0.691, AIC = -184.33) | | |
| SGLT2 mRNA expression in monocytes ^†^ | -0.068 | -0.102 to -0.036 | <0.001 |
| Age | 0.010 | 0.001 to 0.019 | 0.025 |
| FIB-4 index | -0.691 | -0.852 to -0.531 | <0.001 |
| Duration of diabetes ^†^ | -0.033 | -0.112 to 0.046 | 0.406 |
| Waist circumference | -0.002 | -0.012 to 0.008 | 0.712 |
| HbA1c ^†^ | -0.321 | -1.044 to 0.403 | 0.380 |
| eGFR | 0.006 | 0.001 to 0.013 | 0.049 |
| hsCRP | -0.076 | -0.178 to 0.026 | 0.144 |

A multiple linear regression analysis with adjusted beta coefficients. All independent variables corresponded to the values obtained at baseline. The change in FIB-4 index was calculated as the difference between values at 6 months and baseline (FIB-4 index at 6 months − baseline FIB-4 index), with negative values indicating a reduction in the FIB-4 index. Adjusted *R*^2^, adjusted coefficient of determination; AIC, Akaike information criterion; β, partial regression coefficient; CI, confidence interval; eGFR, estimated glomerular filtration rate; FIB-4, fibrosis-4; HbA1c, hemoglobin A1c; hsCRP, high-sensitivity C-reactive protein; SGLT2, sodium-glucose cotransporter 2. ^†^ Log-transformed using the natural logarithm.

**Table S6. Exploratory sensitivity analysis using a fully adjusted multiple linear regression model examining the association between baseline monocyte SGLT2 mRNA expression and FIB-4 index after 6 months of luseogliflozin treatment**

| Dependent variable: FIB-4 index at 6 months | | | |
| --- | --- | --- | --- |
|  | Unstandardized β | 95% CI | *P*-value |
| Exploratory sensitivity analysis | (Adjusted *R*^2^ = 0.432, AIC = -184.33) | | |
| SGLT2 mRNA expression in monocytes ^†^ | -0.068 | -0.102 to -0.036 | <0.001 |
| Age | 0.010 | 0.001 to 0.019 | 0.025 |
| FIB-4 index | 0.309 | 0.148 to 0.469 | <0.001 |
| Duration of diabetes ^†^ | -0.033 | -0.112 to 0.046 | 0.406 |
| Waist circumference | -0.002 | -0.012 to 0.008 | 0.712 |
| HbA1c ^†^ | -0.321 | -1.044 to 0.403 | 0.380 |
| eGFR | 0.006 | 0.001 to 0.013 | 0.049 |
| hsCRP | -0.076 | -0.178 to 0.026 | 0.144 |

A multiple linear regression analysis with adjusted beta coefficients. All independent variables corresponded to the values obtained at baseline. Adjusted *R*^2^, adjusted coefficient of determination; AIC, Akaike information criterion; β, partial regression coefficient; CI, confidence interval; eGFR, estimated glomerular filtration rate; FIB-4, fibrosis-4; HbA1c, hemoglobin A1c; hsCRP, high-sensitivity C-reactive protein; SGLT2, sodium-glucose cotransporter 2. ^†^ Log-transformed using the natural logarithm.
